# Circadian disruption dysregulates lung gene expression associated with inflammatory lung injury

**DOI:** 10.3389/fimmu.2024.1348181

**Published:** 2024-03-14

**Authors:** Nancy G. Casanova, Richard L. De Armond, Saad Sammani, Xiaoguang Sun, Belinda Sun, Carrie Kempf, Christian Bime, Joe G. N. Garcia, Sairam Parthasarathy

**Affiliations:** ^1^ Department of Molecular Medicine, University of Florida Scripps Biomedical Research, Jupiter, FL, United States; ^2^ Department of Medicine, University of Arizona Health Sciences, Tucson, AZ, United States; ^3^ University of Arizona Health Science – Center for Sleep and Circadian Sciences, University of Arizona, Tucson, AZ, United States; ^4^ Department of Pathology, University of Arizona Health Sciences, Tucson, AZ, United States

**Keywords:** circadian rhythm, LPS, gene expression, pathway, cytoskeleton, lung permeability

## Abstract

**Rationale:**

Circadian systems drive the expression of multiple genes in nearly all cells and coordinate cellular-, tissue-, and system-level processes that are critical to innate immunity regulation.

**Objective:**

We examined the effects of circadian rhythm disorganization, produced by light shift exposure, on innate immunity-mediated inflammatory lung responses including vascular permeability and gene expression in a C57BL/6J murine model of inflammatory lung injury.

**Methods:**

A total of 32 C57BL/6J mice were assigned to circadian phase shifting (CPS) with intratracheal phosphate-buffered saline (PBS), CPS with intratracheal lipopolysaccharide (LPS), control (normal lighting) condition with intratracheal PBS, and control condition with intratracheal LPS. Bronchoalveolar lavage (BAL) protein, cell counts, tissue immunostaining, and differentially expressed genes (DEGs) were measured in lung tissues at 2 and 10 weeks.

**Measurements and results:**

In mice exposed to both CPS and intratracheal LPS, both BAL protein and cell counts were increased at both 2 and 10 weeks compared to mice exposed to LPS alone. Multiple DEGs were identified in CPS–LPS-exposed lung tissues compared to LPS alone and were involved in transcriptional pathways associated with circadian rhythm disruption, regulation of lung permeability, inflammation with Rap1 signaling, and regulation of actin cytoskeleton. The most dysregulated pathways included myosin light chain kinase, MAP kinase, profilin 2, fibroblast growth factor receptor, integrin b4, and p21-activated kinase.

**Conclusion:**

Circadian rhythm disruption results in exacerbated immune response and dysregulated expression of cytoskeletal genes involved in the regulation of epithelial and vascular barrier integrity—the mechanistic underpinnings of acute lung injury. Further studies need to explore circadian disorganization as a druggable target.

## Introduction

The circadian system consists of molecular pacemakers that are active in nearly all cells of the body by regulating the expression of “*clock genes*” (1). The peripheral cellular clocks are controlled by the master circadian pacemaker in the suprachiasmatic nucleus of the hypothalamus, which is influenced by various external factors such as light, temperature, and feeding. The clock genes and the circadian system as a whole coordinate a plethora of fundamental cellular-, tissue-, and system-level processes that are critical to regulating immune system function (2, 3). For example, sepsis-related mortality is greater in mice undergoing injury during the transition from light to dark period than the same injury administered during the transition from dark to light period, a finding attributed to circadian influences on gating of cytokine response, immune cell trafficking, and cell numbers (4–6). As such, the immune response to sepsis insult and consequent survival are determined by the state of the mammalian circadian system.

Critically ill patients with sepsis suffer from severe circadian rhythm disruption (7) due to the lack of effective timekeepers in the intensive care unit (ICU) environment due to constant light or varied light–dark (LD) cycle as well as systemic inflammation (8). For example, nuclear factor κB, a central transcription factor mediating immune effector activity, inhibits clock repressors, including the Period, Cryptochrome, and Rev-ERBα genes of the mammalian circadian clock (9). In contrast, REV-ERBα, an important component of the circadian clock, represses the production of proinflammatory cytokines in macrophages (10), supporting the bidirectional relationship between circadian rhythms and inflammation (11). To better understand the mechanisms by which circadian disruption may worsen inflammation, Summa et al. studied gut barrier function in mice with circadian disruption by environmental chronic phase shifts of the LD cycle (circadian disruption similar to “chronic jet lag”) or by genetic mutation of a clock gene (homologous *Clock^ΔD19/D19^*) with superimposed insult from enteral alcohol (12). They posited that gut barrier function would be altered by manipulating the circadian system with chronic jet lag and that the gut barrier function is essential for protection against the proinflammatory intraluminal contents of the gut, such as bacterial endotoxins (i.e., lipopolysaccharide [LPS]) (12). They found that both environmental and genetic circadian disruption promoted alcohol-induced gut leakiness and endotoxemia, possibly through a mechanism involving the tight junction protein in intestinal epithelial cells (Occludin) (12). There exists a knowledge gap as to whether lung barrier dysfunction can be similarly influenced by circadian rhythm disruption. The significance of such a relationship, if present, has important implications for critically ill patients with sepsis and acute lung injury. In critically ill patients with severe infections, acute lung injury is common and associated with increased lung permeability, acute respiratory distress syndrome (ARDS), acute hypoxic respiratory failure, and even death (13, 14). While the direct effect of circulating LPS can certainly be implicated as a causative of acute lung injury, the circadian disorganization observed in critically ill patients residing in ICUs devoid of light–dark cues can potentially confer additional risk (15).

We aimed to determine whether circadian disorganization causes or contributes to increased lung permeability and determine whether induced pathological gene expression is involved. A secondary aim was to determine whether the added insult of LPS-mediated lung injury can accentuate the increased lung permeability and increased pathological gene expression caused by circadian disorganization.

## Materials and methods

### Murine model

All animal procedures were approved by the Institutional Animal Care and Use Committee (IACUC Protocol #17-360). Thirty-two male, 4-week-old, wild-type C57BL/6J mice (JAX, Harborview, ME, USA) were allowed to acclimate for 2 weeks in the environmentally controlled animal facility (12-hour light [0700–1900 hours]:12-hour dark [1900–0700 hours]). At 6 weeks of age, mice were split into two groups of 16 mice and were subjected to circadian phase shifting (CPS) group or remained under control conditions (control group; no-phase shifting [no-PS]).

**Figure 1:**
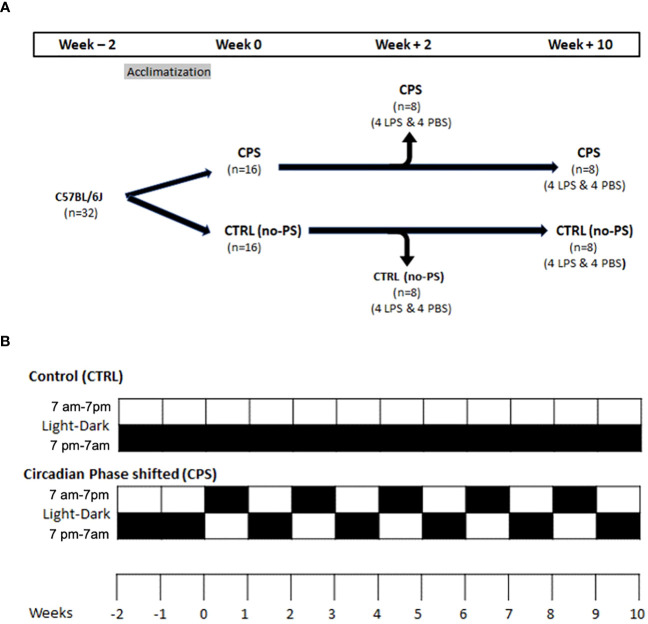
Murine model. **(A)** At 6 weeks of age, mice were split into two groups of 16 mice each: control (no phase shifting [no-PS] or control [CTRL]) and circadian phase shifting (CPS) when the light–dark cycle was shifted weekly for 2 or 10 weeks [as shown in panel **(B)**]. At the end of 2 or 10 weeks, mice underwent tracheal instillation of lipopolysaccharide (LPS) with phosphate-buffered saline (PBS) and were euthanized after 18 hours. **(B)** In the circadian phase shifting (CPS) group, mice were maintained under the standard 12-hour light cycle from 0700 to 1900 hours and then were switched to an alternate light–dark (LD) cycle for 2 and 10 weeks on the Monday of each week (each cell in panel **(B)** represents a week duration). Each box represents 1 week. The lower box is 1900–0700 hours, and the upper box is 0700 to 1900 hours. The mice were subjected to 12-hour light/12-hour dark each day. Black box means a 12-hour dark cycle, and the white box means 12-hour light cycle. No phase-shifted (no-PS; control [CTRL]) mice were maintained on a constant 12-hour:12-hour LD cycle.

In the CPS group (n = 16), mice were subjected to light shift as described below for 2 weeks (n = 8) and 10 weeks (n = 8). The light shift was performed once every 7 days when mice were subjected to 24 hours of light and shifted to an alternate schedule consisting of a 12-hour light cycle from 0700 to 1900 hours and a 12-hour dark cycle from 1900 to 0700 hours. Such an approach has been used by other investigators who have demonstrated the effect of circadian disorganization on increased gut permeability and worsening of atherosclerosis (12, 16, 17). At the end of the 2- or 10-week period of light shift, in each of these subgroups (n = 8), half of the mice (n = 4 in each subgroup) underwent instillation of LPS (Sigma-Aldrich, St. Louis, MO, USA; *Escherichia coli* 0127:B8, 0.1 mg/kg) in 40 µl of 0.9% sterile phosphate-buffered saline (PBS) into the lungs by cannulating the trachea through the vocal cords (**Figure 1**). The remaining half of the mice (n = 4) in each subgroup underwent instillation with PBS alone. Bronchoalveolar lavage (BAL) fluid and lungs were harvested 18 hours post-instillation of LPS or PBS.

In the no-PS (control) group, mice (n = 16) underwent the standard 12-hour light cycle (0700–1900 hours) and 12-hour dark cycle (1900–0700 hours) for 2 weeks (n = 8) and 10 weeks (n = 8). As in the CPS group, in each subgroup (n = 8 mice), half of the mice (n = 4) underwent instillation of LPS+PBS into the lungs by cannulating the trachea through the vocal cords, and half of the mice underwent instillation with the PBS alone (**Figure 1**). BAL fluid and lungs were harvested 18 hours post-instillation of LPS or PBS. Mouse lungs were excised and embedded for hematoxylin and eosin staining.

Immunohistochemistry (IHC) studies were performed on paraffin-embedded sections with antibodies for non-muscle myosin light chain kinase (MYLK), which are specific for mouse, rat, and human N-terminus of MYLK (sc-365352, Santa Cruz Biotechnology, Dallas, TX, USA).

Mice were anesthetized by intraperitoneal injection of ketamine (100 mg/kg) and xylazine (5 mg/kg) for the tracheal intubation and LPS instillation. After 18 h, mice were re-anesthetized with ketamine (100 mg/kg) and xylazine (5 mg/kg) and then euthanized by exsanguination. Details regarding the LPS-induced acute lung injury murine model, BAL procedure, lung tissue RNA extraction, RNA sequencing, and euthanasia are provided in the online supplement in a manner identical to prior studies (18–27).

### Statistical analysis

Continuous data were compared using non-parametric methods and categorical data by χ-square test. Where applicable, standard one-way ANOVA and t-test were used to compare the mean from two or more experimental groups. Differences between groups were considered statistically significant when p < 0.05. All analyses were performed using Stata v. 17 (StataCorp, TX, USA), and GraphPad Prism v. 8.0 software (San Diego, CA, USA).

## Results

### CPS exacerbates LPS-induced increases in lung inflammation and vascular permeability

**Figure 2:**
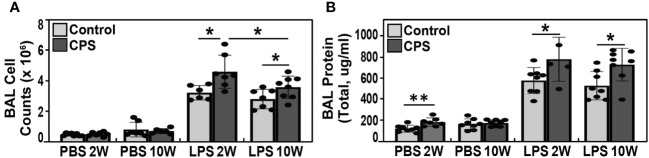
BAL cell count and protein levels. **(A)** Bronchoalveolar lavage (BAL) total cell counts at 2 and 10 weeks of circadian phase shifting (CPS) in lipopolysaccharide (LPS)-treated C57BL/6J mice (N = 5–8). We observed a significant increase in the cell counts when LPS was instilled in addition to the CPS exposure, with the higher cell counts observed at 2 weeks when compared to 10 weeks (p-value <0.05). **(B)** BAL total protein after 2 weeks of CPS was significantly higher when compared to mice in the no-PS group (p-value <0.005), but there were no differences in BAL protein levels at 10 weeks of CPS versus non-PS groups (p-value 0.64 and 0.5, respectively). The BAL protein levels in LPS-exposed mice were significantly greater in the CPS group when compared to non-PS group at both 2 and 10 weeks (p-value 0.01 and 0.001, respectively). Similar to the BAL cell count, the highest total protein level was observed at 2 weeks. * p value <0.05, ** p value 0.005.

There were no differences in the cell counts at 2 weeks or 10 weeks observed between the CPS and no-PS groups (p = 0.64 and p = 0.5, respectively) (**Figure 2A**). Similarly, at 10 weeks, the CPS+LPS combination resulted in significantly greater cell counts and BAL protein when compared to receiving LPS alone (p = 0.004 and 0.001, respectively; **Figures 2A, B**). After 2 weeks of exposure to light shift, a significant increase in BAL protein was observed in the CPS group (PBS 2W) compared to the no-PS group (p = 0.005). In contrast, this difference between CPS and no-PS groups was no longer present at 10 weeks (p = 0.6) (**Figure 2B**). The combination of CPS and LPS exposures produced significantly increased BAL protein and cell counts at 2 weeks compared to only the LPS challenge (p = 0.01 and 0.01, respectively). Interestingly, the cell counts at 10 weeks in the CPS+LPS group were significantly decreased compared to those at 2 weeks in the same CPS+LPS group (p = 0.05) (**Figure 2A**). However, the mean levels of BAL protein were not different between 2 and 10 weeks in the CPS+LPS group (p = 0.6) (**Figure 2B**).

### Effect of circadian phase shifting on transcriptome response

**Figure 3:**
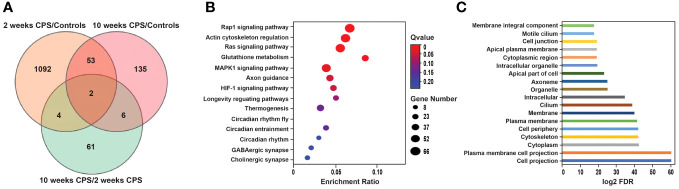
Circadian phase shifting (CPS)-associated differential gene expression and enrichment analysis. **(A)** The total number of differentially expressed genes (DEGs) (false discovery rate (FDR) < 0.005) in CPS compared to controls (no-PS) at weeks 2 and 10 and the DEGs after CPS at 2 weeks versus 10 weeks of CPS exposure. Venn diagram shows the overlap of the differentially expressed genes in the CPS *vs.* controls at 2 (1,151 DEGs) and 10 weeks (196 DEGs) and the CPS overtime comparison (73 DEGs). Orange represents the DEGs at 2 weeks of CPS compared to controls; pink indicates the DEGs at 10 weeks of CPS compared to controls; green shows the DEGs at 10 weeks of CPS compared to 2 weeks of CPS. **(B)** Bubble chart of the Kyoto Encyclopedia of Genes and Genomes (KEGG) pathway enrichment of the DEGs after 2 weeks of CPS compared to controls (no-phase shift). Rap1 and the regulation of actin cytoskeleton were the pathways with more genes annotated. X-axis is the enrichment ratio (number of genes annotated to an entry in the selected gene set to the total number of genes annotated to the entry in the species), and Y-axis is KEGG pathway. The size of the bubble correlates with the number of genes annotated to the pathway; the red color represents the ones with enriched significance (Q value >0.05). **(C)** Gene Ontology of the top 18 cellular components enriched at 2 weeks of CPS and ranked according to their level of significance. X-axis indicates the log2 FDR. Gene Ontology (GO) top terms revealed the cellular structures associated with the dysregulated genes; among them were the cytoskeleton and various associated plasma membranes and cell junctions.

Transcriptome responses to CPS at 2 and 10 weeks against duration-matched control groups revealed that CPS triggers transcriptional changes in a larger number of differentially expressed genes (DEGs) at 2 weeks compared to CPS at 10 weeks. Compared to controls, a total of 1,151 DEGs (false discovery rate (FDR) <0.05) were present after exposure to CPS for 2 weeks, contrasting with only 196 DEGs (FDR < 0.05) present at 10 weeks of CPS. The total number of DEGs in CPS compared to controls (no-PS) at weeks 2 and 10 and the DEGs after CPS at 2 weeks versus 10 weeks of CPS exposure are depicted in **Figure 3A**.

Of the 1,151 DEGs at 2 weeks of CPS, 624 genes were upregulated and 527 downregulated. Among the top DEGs, 32 genes observed a change in their expression above log FC |1|. These genes are presented in **Table 1**. *Mylk4* encoding a myosin light chain kinase, involved in protein phosphorylation and an integral component of membrane, and *Mapk10*, another kinase involved in proliferation, differentiation, and transcriptional regulation, were among the top downregulated genes. In contrast, *Tecte3*, or Dynein Light chain (DYNLT2), and *Shisa12A*, a transmembrane adaptor protein, an integral component of the membrane, were the most upregulated genes. Our enrichment analysis incorporated all the DEGs with FDR < 0.05. The pathway analysis (**Figure 3B**) identified Rap1 signaling, an important regulator of integrins and cadherins for cell adhesion, and the regulation of actin cytoskeleton among the pathways with higher enrichment ratio, based on genes annotated (26 genes). In addition, our results revealed that the CPS for 2 weeks affected genes primarily involved in three circadian-related biological pathways: circadian, circadian-fly, and circadian entrainment. Other dysregulated pathways included genes involved in the GABAergic synapse and HIF-2 signaling pathways. HIF-2 is an essential mediator of the cellular-oxygen signaling involved in cell proliferation, angiogenesis, metabolism, and cancer (28). We next conducted a Gene Ontology (GO) overview for cellular components for the DEGs, yielding 81 terms; of those, the top 18 (FDR < 4.35E−06) revealed cellular structures in which these gene products perform their functions: cell projection, plasma membrane, cytoskeleton, cilium, and cell junction (**Figure 3C**).

**Table 1:** Top DEGs at 2 weeks of circadian phase shifting.

Gene name	Log2 fold change	p adjusted value
LOC102639505	−5.56E+00	2.36E−02
Prg2	−3.05E+00	4.16E−02
Xirp2	−2.39E+00	1.55E−03
Mlip	−2.12E+00	3.95E−02
Hspa1a	−2.09E+00	3.50E−02
Hspa1b	−1.99E+00	2.11E−02
Mapk10	−1.73E+00	1.51E−02
Ptpn5	−1.68E+00	2.11E−02
Spta1	−1.44E+00	1.91E−02
Mylk4	−1.36E+00	3.66E−02
Lama1	−1.27E+00	1.24E−03
LOC100041504	−1.15E+00	1.54E−02
Disp2	−1.02E+00	4.27E−06
Tcte3	5.83E+00	1.00E−02
Shisal2a	2.17E+00	3.93E−02
Gm2808	1.79E+00	6.20E−03
2010109I03Rik	1.58E+00	1.43E−02
Pramef12	1.39E+00	2.61E−02
Synb	1.36E+00	4.51E−02
Nr1i2	1.26E+00	1.51E−02
Unc79	1.25E+00	4.16E−02
Orm2	1.25E+00	3.30E−02
Necab2	1.24E+00	3.75E−02
Tctex1d4	1.21E+00	2.51E−05
Thegl	1.20E+00	9.01E−03
Ccdc148	1.19E+00	1.10E−03
Aoc1	1.18E+00	2.55E−05
1700056E22Rik	1.13E+00	2.86E−02
Slc35g3	1.12E+00	4.05E−03
Fer1l4	1.07E+00	1.48E−02
Plk5	1.06E+00	4.66E−02
Apobec4	1.03E+00	3.75E−02
Ttll8	1.01E+00	2.46E−02

DEGs, differentially expressed genes.

### CPS induces dysregulation of actin cytoskeleton pathway genes: *Mylk4* and multiple integrin genes

**Figure 4:**
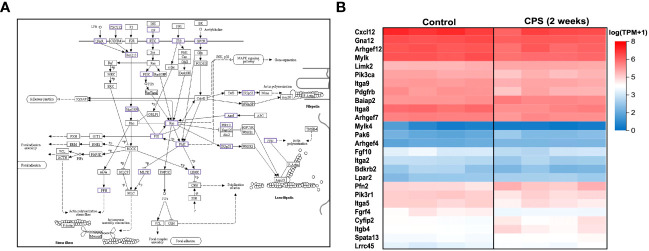
Circadian phase shifting (CPS) and the cytoskeleton pathway. **(A)** Kyoto Encyclopedia of Genes and Genomes (KEGG) pathway map of the regulation of actin cytoskeleton (Kanehisa Laboratories, adapted with permission). The dysregulated genes associated with CPS are highlighted in color purple. β_1_ integrin and FAK regulate cell migration by controlling dynamics of focal adhesions and the actin cytoskeleton. Myosin light chain kinase (MYLK) phosphorylation modulates cell contraction to facilitate smooth muscle cell migration. **(B)** Heatmap of the differentially expressed genes at 2 weeks of CPS compared to controls. For the 26 differentially expressed genes (DEGs) contained within the cytoskeleton pathway, red indicates upregulated, and blue is downregulated. Pfn2, Fgfr4, Itgb4, Mylk4, Bdkrb2, Pak6, and Arhef4 were the most significant (FDR 0.05, fold change 0.05). We observed subtle gene expression differences between the controls and the CPS at 2 weeks, which do not exhibit directionality changes in the expression, based on the log (TPM+1) values.

Unbiased analysis of the differential expression of the genes after 2 weeks of CPS included genes that were associated with the regulation of the actin cytoskeleton pathway. A total of 26 DEGs were contained in the cytoskeleton pathway. **Figure 4A** highlights the DEGs and their role in the actin cytoskeleton regulation showing the close interactions with the focal adhesion and MAPK pathways; all these are of critical importance for the endothelial pulmonary vascular barrier. The heatmap in **Figure 4B** depicts the subtle differences in gene expression observed after 2 weeks of CPS; among the genes annotated in the cytoskeleton pathway, *Pfn2*, *Fgfr4*, *Itgb4*, *Mylk4*, *Bdkrb2*, *Pak6*, and *Arhef4* achieved higher fold change (>0.5). Endothelial contractile events are regulated by the phosphorylation of regulatory myosin light chains catalyzed by Ca(2+)/calmodulin-dependent myosin light chain kinase (*Mylk*); this phosphorylation modulates cell contraction to facilitate smooth muscle cell migration (29). Our results observed a limited number of DEGs at 10 weeks of CPS (196), limiting our enrichment analysis, and no signaling related to cytoskeletal regulation, with most of the DEGs contained in metabolic pathways.

### CPS induces inflammation and changes in *Mylk* expression in lung tissue

**Figure 5:**
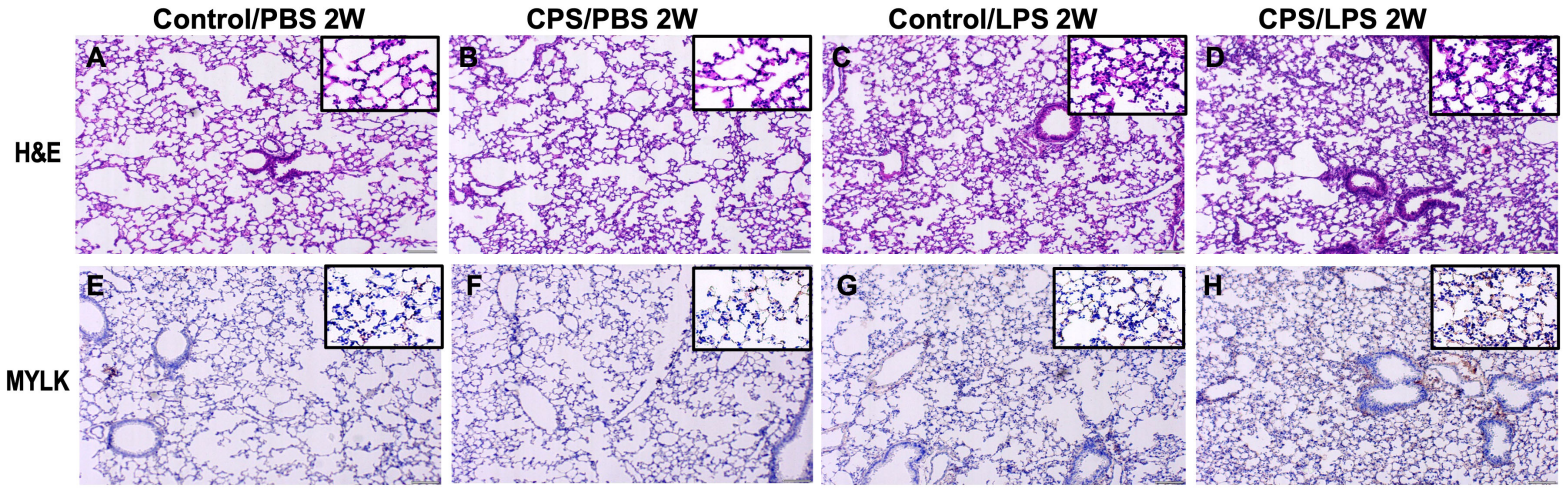
Histology with H&E staining and immunohistology of MYLK in murine model. Mice were exposed to 2 weeks of circadian phase shifting (CPS) **(B, D, F, H)** or no shifting (control) **(A, C, E, G)** and injected with lipopolysaccharide (LPS) 0.1 mg/kg **(C, D, G, H)** or phosphate-buffered saline (PBS) **(A, B, E, F)**. Mice were harvested, lung tissue sections were stained with hematoxylin and eosin (H&E) **(A–D)**, and immunohistochemistry (IHC) studies were performed with antibodies for MYLK **(E–H)** (×200). H&E staining of lung tissues from mice exposed to LPS shows acute lung inflammation with intra-alveolar neutrophil infiltration in both CPS and control, especially in CPS mice. IHC staining of LPS-exposed lung tissues exhibited increases in MYLK immunoreactivity (brown) and further significant increases in CPS group, compared with controls (main ×200, inset ×400).

Histological and immunohistochemistry studies were designed to study myosin light chain kinase (Mylk), a key regulator of myosin–actin filament phosphorylation in the cytoskeletal transcendent during the inflammatory response (e.g., apoptosis, vascular permeability, and leukocyte diapedesis), cell motility and morphology, and airway hyperresponsiveness. In lung tissue of mice exposed to CPS, a significantly increased acute lung inflammation was observed in CPS for 2 weeks, compared to control (non-CPS) (**Figures 5A–D**). IHC staining of mouse lungs with anti-Mylk indicated that LPS significantly increased Mylk immunoreactivity in both CPS and non-CPS at 2 weeks, with more Mylk in the CPS group than in the non-CPS group (**Figures 5E–H**). Similarly, H&E staining and immunohistology, as well as the levels of MYLK, were robustly increased in LPS/control and further significantly increased (p < 0.05) when CPS was added at 10 weeks (**Supplementary Figures S2**, **S3**).

### Circadian phase shifting combined with LPS potentiates dysregulation of immunological pathways

**Figure 6:**
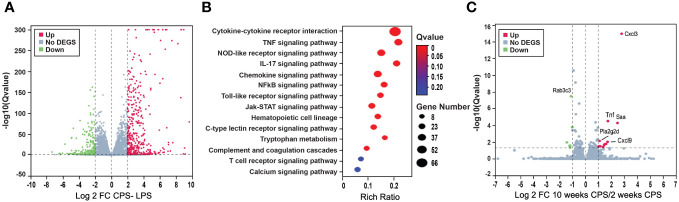
Circadian phase shifting (CPS) combined with lipopolysaccharide (LPS) potentiates dysregulation of genes. **(A)** Volcano plot shows the differentially expressed genes (DEGs) after LPS–CPS, combined treatment. A slightly higher number of dysregulated genes were observed compared to LPS alone without CPS, with a similar number of DEGs (false discovery rate (FDR) 0.05 and FC 2) at week 2, with 632 and 636, respectively, and at week 10, there were 660 DEGs with combined insult and 567 DEGs with LPS alone. **(B)** Pathway analysis for LPS–CPS combined treatment at 2 weeks, highlighting immune and inflammatory pathways that remained altered at 10 weeks: cytokine–cytokine receptor interaction, TNF signaling, and NOD-like receptor signaling. **(C)** CPS at 10 and 2 weeks. Volcano plot denoting the differential gene expression at 2 weeks of CPS compared to 10 weeks of CPS: in red are the upregulated genes and in green are the downregulated genes. Highlighted as the topmost upregulated genes chemokines are Cxcl3, Cxcl9, Tnf, and Pla2g2d. Rab3c was the most downregulated gene.

LPS treatment released an intense transcriptome response characterized by a high number of DEGs with dysregulation of primarily immunological-related pathways. When LPS treatment was combined with CPS, a slightly higher number of dysregulated genes were observed than LPS without the CPS, with a similar number of DEGs (FDR of 0.05 and FC 2) at week 2, with 632 and 636, respectively; at week 10, there were 660 DEGs with combined insult and 567 DEGs with LPS alone. Pathway signaling in both treatment categories at 2 and 10 weeks highlighted immune and inflammatory pathways that remained altered at 10 weeks: cytokine–cytokine receptor interaction, TNF signaling, and NOD-like receptor signaling (**Supplementary Table S1**). It was observed that the response to CPS was masked by the intense immunological response derived from the LPS treatment, represented by the minimal differences between LPS alone and LPS+CPS groups, with only 23 DEGs (FDR < 0.05, FC 2). All DEGs were primarily related to the innate immune and antibacterial activities triggered by LPS (**Figures 6A, B**).

To gain further insights into the underlying mechanisms of time-based abrogation, we conducted a comparative analysis of CPS responses at two different time points: week 10 and week 2. Our analysis revealed a total of 73 DEGs with an FDR below 0.05 (**Supplementary Table S2**). Among these DEGs, we observed significant upregulation of chemokines Cxcl3 and Cxcl9, as well as Tnf, which are known to be crucial regulators of the proinflammatory response and innate immune system (**Figure 6C**). Another prominently dysregulated gene, Pla2g2d, a phospholipase, was also upregulated. In contrast, Rab3c, a RAS oncogene family, was the most downregulated gene.

## Discussion

In a well-established model of lung permeability, we found that circadian disorganization increased lung permeability in mice and worsened LPS-induced lung permeability. We found that disorganization of circadian structure led to increased pathological gene expression associated not only with immunological responses but also central to the regulation of lung permeability. We assessed lung permeability by measuring total protein and the number of immune cells present in BAL samples, as elevated total protein and polymorphonuclear leukocytes in the BAL correlate with gold standard measures of lung permeability (30). At 2 weeks, circadian disorganization induced by the phase shifting of the LD cycle—without the additional insult from LPS—produced a significant increase in BAL protein and cell count levels. The addition of LPS insult to the circadian disorganization evoked the highest levels of protein and cell counts in the BAL at both 2 and 10 weeks. These findings are similar to prior reports of increased intestinal permeability observed by circadian disorganization induced by phase shifting of the LD cycle (12). However, to our knowledge, this is the first report of increased lung permeability due to circadian disorganization. Summa et al. found that the worsening of intestinal permeability due to circadian disorganization was further worsened by the additional insult of alcohol ingestion (12). Similarly, in our study, the co-administration of LPS led to worsening lung permeability, as evidenced by greater total protein and polymorphonuclear leukocytes in BAL of mice receiving LPS and circadian disorganization when compared to LPS alone. These findings support the premise that the circadian clock controls pulmonary inflammation (31) and indicates that circadian disorganization may be an unrecognized risk factor for acute lung injury. In humans with septic shock, circadian rhythms are known to be disorganized or delayed in phase (7, 32–34). Such circadian disorganization may further worsen lung permeability in addition to circulating LPS in the presence of infection and thereby contribute to the development of acute lung injury in critically ill patients. Genetic ablation of the clock gene Bmal1 (also called Arntl or MOP3) in bronchiolar cells disrupts rhythmic Cxcl5 expression, resulting in exaggerated inflammatory responses to LPS (31). As circadian rhythm disturbances during the first 2 weeks appear to contribute to the pathobiology of acute lung injury, this may represent a reversible therapeutic target. We observed significant temporal variations in gene expression patterns, particularly evident at week 2 of CPS, as compared to the 10-week time point with pronounced differential expression of genes associated with proinflammatory cytokines and innate immune system regulation (Cxcl3, Cxcl9, Saa, and TNF). Comparatively, the reduced number of DEGs at 10 weeks of CPS indicates acclimation. BAL cell count differences between weeks 2 and 10 reflect the LPS-induced inflammatory response, supported by extensive differential gene expression. Conversely, BAL cell counts show no significant differences with CPS alone, paralleled by subtle transcriptome changes at 2 weeks and even more reduced DEGs at 10 weeks. Significantly elevated BAL protein levels at week 2, not at week 10, align with a higher number of DEGs at week 2 of CPS compared to CPS at 10 weeks, highlighting the correlation between cellular responses and gene expression patterns. Additionally, we observed altered expression of phospholipases, specifically Pla2g2d, which is known to play a critical role in T-cell differentiation, proliferation, and activation. Interestingly, research suggests that Pla2g2d knockout (KO) mice exhibit diminished lung damage, implicating its involvement in susceptibility to severe acute respiratory syndrome (35). Another gene of interest is serum amyloid A (Saa), which has been shown to confer a pathogenic role by promoting proinflammatory Th17 cell differentiation and directly influencing T cells in collaboration with STAT3 (36). Proinflammatory TNF is involved in sleep regulation and orchestrates phosphorylation events in microglial cells (37) and lung endothelial cells through the regulation of cytozymes like NAMPT and activation of NF-kB (19, 38). Similarly in myeloid cells, the circadian variations in *LysM^Bmal1+/+^* mice exposed to ventilator-induced lung injury were associated with increased inflammatory response and severity (39).

Interestingly, a noticeable attenuation of lung permeability at 10 weeks of continued circadian disorganization was observed (**Figure 1**), suggesting an adaptive response to attenuate the circadian disorganization-induced increases in lung permeability. The attenuated gene expression at 10 weeks of CPS substantiates such adaptive mechanisms. Specifically, it was observed that compared to matched controls, only 196 DEGs were present at 10 weeks of CPS in contrast to the 1,151 DEGs observed at DEGs at 2 weeks, substantiating the activation of an adaptive mechanism that occurs at the gene expression level. Other studies have shown that changes in the transcriptome are not only tissue-specific but also time-related, with higher expression observed at the beginning of the intervention and lower expression toward the end of the intervention period (40). Microarray expression also indicated differences around tissue specificity; sleep–circadian rhythm dysregulation induced a larger number of dysregulated genes in the liver than in the brain after 6 hours; similarly, the overlap of blood transcriptomic between 1 week of sleep–circadian rhythm dysregulation and the transcripts in mouse liver after 2 week was only 46% (40).

Disruption of the circadian rhythm alters the transcriptome, leading to changes in the metabolism and cellular response that result in deleterious health outcomes (11, 41). In the study of intestinal permeability, Summa et al. found significant increases in cytoplasmic Occludin—an important tight junction protein—which was internalized by genetic (CLOCK gene mutation) and alcohol-induced changes and led to loss of gut barrier function. In our study, transcriptomic changes caused by CPS in lung tissue revealed dysregulation of the actin cytoskeleton, a mechanosensory process that signals changes in actin polymerization dynamics via exposure to hidden protein binding sites that alter actin-binding protein interactions (21). Our results indicate that 2 weeks of CPS alters the cytoskeleton integrity at the gene expression level, of critical importance for the endothelial pulmonary vascular barrier homeostasis, therefore altering the lung’s ability to restrict fluid and protein in the vascular space at normal vascular pressures and protecting lung alveoli from lethal flooding, which are mechanisms altered in acute lung injury. Current concepts of lung vascular barrier regulation involve highly dynamic lung endothelial cell barrier regulation by the actomyosin cytoskeleton, which choreographs spatially directed increases in cellular tension, which can either favor barrier-disruptive contractile forces, thus promoting vascular permeability, or, conversely, favor barrier-protective tethering forces, driven by cytoskeletal proteins, which promote barrier stabilization and restoration of the intact endothelial cell barrier during recovery (29, 42). The endothelial contractile apparatus is regulated by the phosphorylation of regulatory myosin light chains catalyzed by the Ca(2+)-dependent myosin light chain kinase. We identified that *Mylk4* encoding myosin light chain kinase 4 was among the most downregulated genes at 2 weeks of CPS. *Mylk4* is an integral component of the focal adhesion pathway involved in protein phosphorylation, with a key role in the altered cytoskeletal network of cardiomyocytes in heart failure and malignant cell proliferation (43, 44). *MYLK* encodes protein isoforms involved in multiple components of the inflammatory response, including apoptosis, vascular permeability, and leukocyte diapedesis, and is a well-established candidate gene in sepsis- or trauma-induced ARDS (45, 46). Our results identified Tecte3 and Tctex14 (dynein light chains) among the most highly upregulated genes after CPS, and both are key modulators of the microtubule cytoskeleton. Located in the cytosol, Tecte3 is associated with primary ciliary dyskinesia and diaphragmatic disease (47). Similarly, at 2 weeks, we observed multiple differentially expressed integrins that serve as membrane receptors that mediate cell–matrix or cell–cell adhesion and transduce signals that control dynamics of focal adhesions and the actin cytoskeleton. Integrins identified include Itga9 (receptor for VCAM1, cytoactin, and osteopontin), Itga8, Itgb4, and Itga2 (receptors for laminin) and Itga5 (receptor for fibrinogen) and were annotated within multiple pathways such as the regulation of actin cytoskeleton, β_1_ integrin, and FAK. Pak proteins were altered at 2 weeks of CPS and are critical effectors that link RhoGTPases to cytoskeleton reorganization and MAPK signaling pathway activation. Thus, circadian disorganization-induced DEGs reflect biological plausibility in contributing to phase shifting of the LD cycle on lung permeability.

Our study exhibits several limitations including but not accounting for possible circadian variations that may occur in BAL collected at different time points. Further studies are needed to verify the extent of the CPS in lung physiology.

In conclusion, our study uncovered that circadian disorganization not only impacts circadian pathways but also disrupts actin cytoskeletal regulation, a critical mechanism for preserving lung vascular integrity. Genes that regulate critical cytoskeletal effectors are intimately involved in preserving lung barrier function, and their dysregulation can lead to the observed increased lung permeability. In sum, our study showed that circadian disorganization caused increased lung permeability and increased pathological gene expression associated with increased lung permeability. When superimposed with LPS-mediated lung injury, lung permeability is further exacerbated. The significance of these findings holds critical implications for critically ill patients with acute lung injury. While the direct effect of circulating LPS in critically ill patients with infections can certainly be implicated as causative of acute lung injury, the circadian disorganization observed in such patients residing in an ICU with disruptions in light–dark cycles can potentially confer additional risk and serve as a potential target for improving lung inflammation.

## Data availability statement

The data presented in the study are deposited in the SRA NCBI repository, accession number PRJNA1083044.

## Ethics statement

The animal study was approved by Institutional Animal Care and Use Committee (IACUC Protocol# 17-360). The study was conducted in accordance with the local legislation and institutional requirements.

## Author contributions

NC: Conceptualization, Data curation, Formal Analysis, Investigation, Methodology, Project administration, Resources, Software, Validation, Visualization, Writing – original draft, Writing – review & editing. RD: Data curation, Investigation, Methodology, Project administration, Validation, Visualization, Writing – review & editing. SS: Data curation, Formal Analysis, Investigation, Methodology, Project administration, Software, Writing – review & editing. XS: Data curation, Methodology, Software, Writing – review & editing. BS: Data curation, Methodology, Writing – review & editing. CK: Data curation, Methodology, Investigation, Writing – review & editing. CB: Validation, Resources, Writing – review & editing. JG: Conceptualization, Funding acquisition, Investigation, Resources, Supervision, Writing – original draft, Writing – review & editing. SP: Conceptualization, Funding acquisition, Investigation, Project administration, Resources, Supervision, Validation, Writing – original draft, Writing – review & editing.

## Supplementary material

Supplementary Table 1 The top 25 Kegg pathways from the DEGs in the LPS and LS combined insult.

Supplementary Table 2 The top differentially expressed genes between the CPS at week 2 and CPS at week 10.

Supplementary Figure 1 Histology H&E staining and immunohistology of MYLK at 2 weeks of exposure to CPS. Same as **Figure 5** but inset 400 X.

Supplementary Figure 2 Histology with H&E staining and immunohistology of MYLK in murine model. Mice were exposed to 10 weeks of circadian phase shifting (CPS) **(B, D, F, H)** or no shifting (Control) **(A, C, E, G)** and injected with LPS 0.1 mg/kg **(C, D, G, H)** or PBS**(A, B, E, F)**. Mice were harvested, and lung tissue sections stained by Hematoxylin and Eosin (H&E) **(A-D)** and immunohistochemistry studies (IHC) performed with antibodies for MYLK **(E–H)** (×200). H&E staining of lung tissues from mice exposed to LPS show acute lung inflammation with intra-alveolar neutrophil infiltration in both CPS and Control, especially in CPS mice. IHC staining of LPS-exposed lung tissues exhibited increases in MYLK immunoreactivity (brown), and further significant increases in CPS group, compared with Controls (main 200X).

Supplementary Figure 3 Quantification of Histology with H&E staining and immunohistology of MYLK in 2 weeks and 10 weeks of circadian phase shifting murine model. **(A)** H&E staining area significantly increased in control/LPS mice (*p<0.05 vs. controls), and further increased in CPS/LPS with CPS for 2 weeks and 10 weeks (**p<0.05 vs. control/LPS). **(B)** IHC staining of LPS-exposed lung tissues exhibited increases in MYLK immunoreactivity (*p<0.05 vs. controls), and further significant increases in CPS/LPS with CPS for 2 weeks and 10 weeks (**p<0.05 vs. control/LPS).
