# Supplementary material for: Circadian disruption dysregulates lung gene expression associated with inflammatory lung injury
Source: Front Immunol. 2024 Mar 14;15:1348181. doi: 10.3389/fimmu.2024.1348181 (PMC10979643; doi:10.3389/fimmu.2024.1348181)
Supplement: Supplementary Table 1 — The top 25 Kegg pathways from the DEGs in the LPS and LS combined insult. [file Table_1.docx]

| **The top 25 Kegg pathways from the DEGs in the LPS and LS combined insult.** | | | |
| --- | --- | --- | --- |
| KEGG Pathway Term Desc | Genes contained | P value | Q value |
| Cytokine-cytokine receptor interaction | 66 | 1.12E-36 | 1.96E-34 |
| TNF signaling pathway | 31 | 3.83E-18 | 3.35E-16 |
| NOD-like receptor signaling pathway | 31 | 1.73E-13 | 8.92E-12 |
| IL-17 signaling pathway | 23 | 2.04E-13 | 8.92E-12 |
| Chemokine signaling pathway | 30 | 5.42E-12 | 1.90E-10 |
| NF-kappa B signaling pathway | 20 | 1.15E-09 | 3.36E-08 |
| Toll-like receptor signaling pathway | 16 | 2.09E-07 | 5.23E-06 |
| Jak-STAT signaling pathway | 20 | 4.84E-07 | 1.06E-05 |
| Hematopoietic cell lineage | 16 | 5.76E-07 | 1.12E-05 |
| C-type lectin receptor signaling pathway | 15 | 6.32E-06 | 1.11E-04 |
| Tryptophan metabolism | 8 | 1.13E-04 | 0.001799 |
| Cytosolic DNA-sensing pathway | 9 | 2.11E-04 | 0.00307 |
| Osteoclast differentiation | 13 | 4.38E-04 | 0.005898 |
| Neuroactive ligand-receptor interaction | 20 | 0.001703 | 0.020167 |
| Complement and coagulation cascades | 10 | 0.001729 | 0.020167 |
| RIG-I-like receptor signaling pathway | 7 | 0.008631 | 0.094405 |
| Phagosome | 14 | 0.011102 | 0.114283 |
| Th17 cell differentiation | 9 | 0.013591 | 0.132131 |
| T cell receptor signaling pathway | 8 | 0.022959 | 0.211469 |
| Calcium signaling pathway | 12 | 0.026449 | 0.230486 |
| Phenylalanine, tyrosine and tryptophan biosynthesis | 2 | 0.030947 | 0.230486 |
| Penicillin and cephalosporin biosynthesis | 1 | 0.03161 | 0.230486 |
| D-Arginine and D-ornithine metabolism | 1 | 0.03161 | 0.230486 |
| Intestinal immune network for IgA production | 5 | 0.033478 | 0.234343 |

DEGs, differentially expressed genes
