# Supplementary figures and images for: Circadian disruption dysregulates lung gene expression associated with inflammatory lung injury
Source: Front Immunol. 2024 Mar 14;15:1348181. doi: 10.3389/fimmu.2024.1348181 (PMC10979643; doi:10.3389/fimmu.2024.1348181)

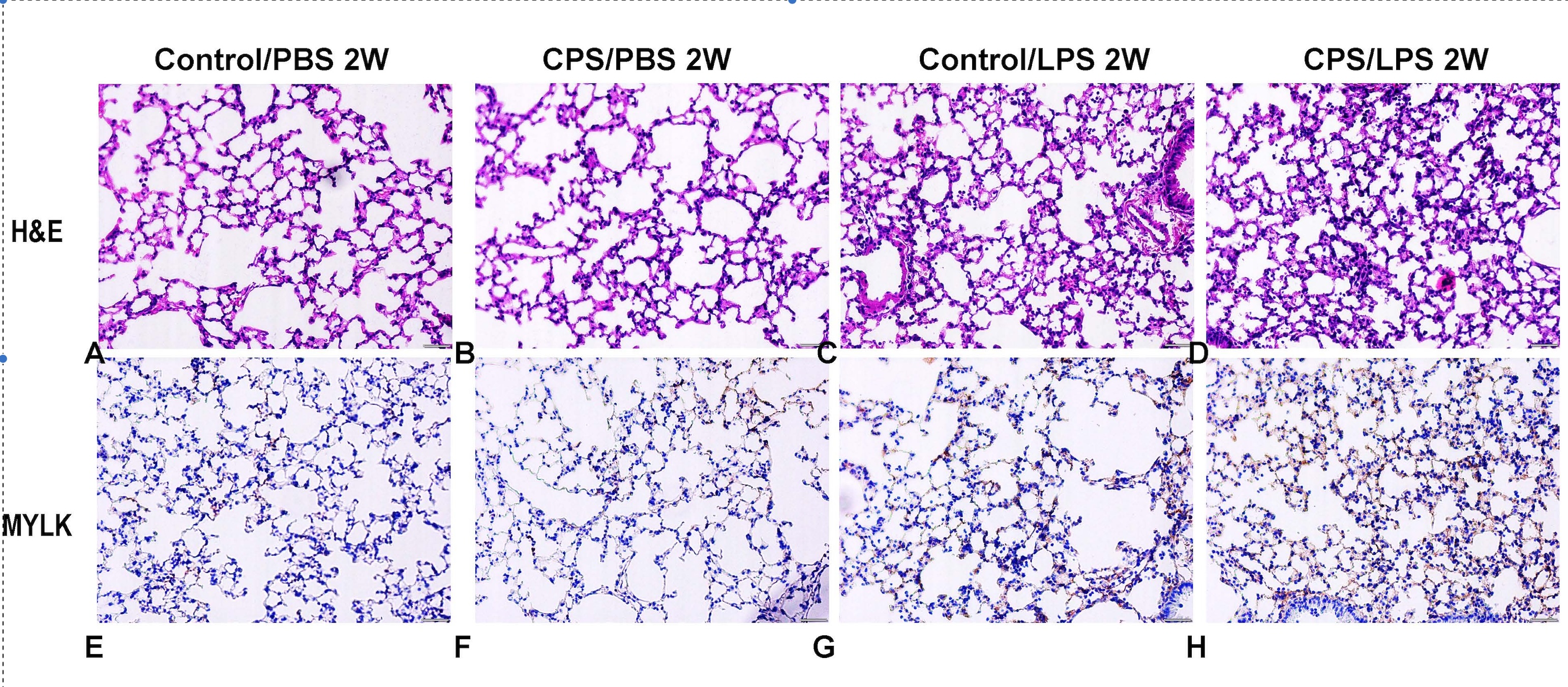

Supplement: Supplementary Figure 1 — Histology H&E staining and immunohistology of MYLK at 2 weeks of exposure to CPS. Same as Figure 5 but inset 400 X. [file Image_1.jpeg]

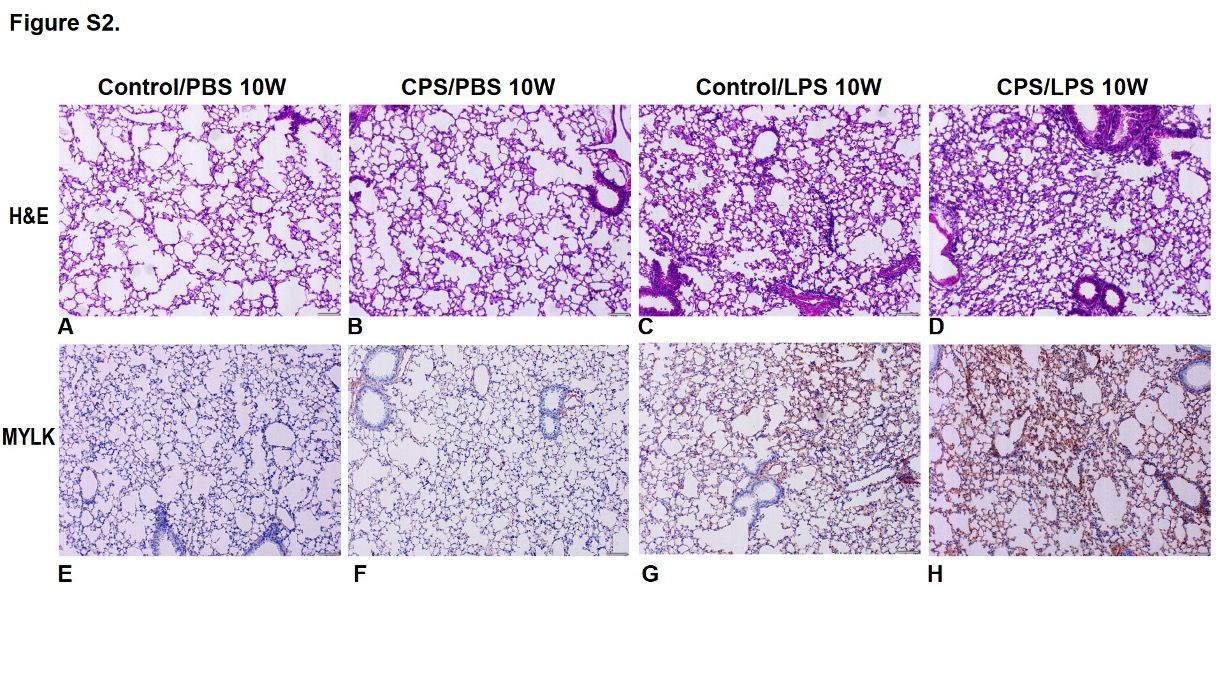

Supplement: Supplementary Figure 2 — Histology with H&E staining and immunohistology of MYLK in murine model. Mice were exposed to 10 weeks of circadian phase shifting (CPS) (B, D, F, H) or no shifting (Control) (A, C, E, G) and injected with LPS 0.1 mg/kg (C, D, G, H) or PBS(A, B, E, F). Mice were harvested, and lung tissue sections stained by Hematoxylin and Eosin (H&E) (A-D) and immunohistochemistry studies (IHC) performed with antibodies for MYLK (E–H) (×200). H&E staining of lung tissues from mice exposed to LPS show acute lung inflammation with intra-alveolar neutrophil infiltration in both CPS and Control, especially in CPS mice. IHC staining of LPS-exposed lung tissues exhibited increases in MYLK immunoreactivity (brown), and further significant increases in CPS group, compared with Controls (main 200X). [file Image_2.jpeg]

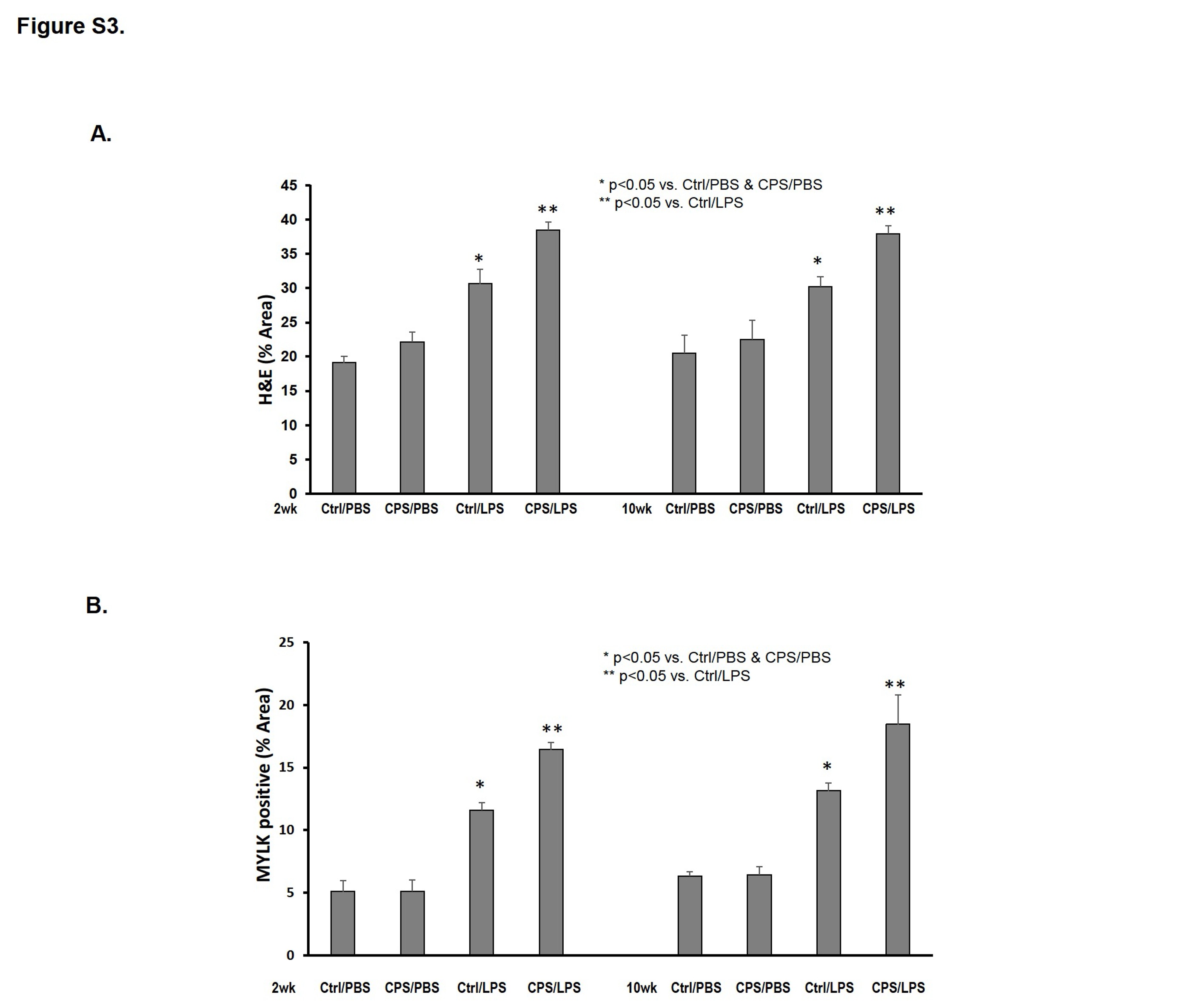

Supplement: Supplementary Figure 3 — Quantification of Histology with H&E staining and immunohistology of MYLK in 2 weeks and 10 weeks of circadian phase shifting murine model. (A) H&E staining area significantly increased in control/LPS mice (*p<0.05 vs. controls), and further increased in CPS/LPS with CPS for 2 weeks and 10 weeks (**p<0.05 vs. control/LPS). (B) IHC staining of LPS-exposed lung tissues exhibited increases in MYLK immunoreactivity (*p<0.05 vs. controls), and further significant increases in CPS/LPS with CPS for 2 weeks and 10 weeks (**p<0.05 vs. control/LPS). [file Image_3.jpeg]
